# Supplementary material for: Neuid: A Novel Neuron‐Enriched LncRNA that Connects Epigenetic Gene Silencing to Alzheimer's Disease
Source: Adv Sci (Weinh). 2026 Mar 14;13(29):e14972. doi: 10.1002/advs.202514972 (PMC13205658; doi:10.1002/advs.202514972)
Supplement: Supplementary file 1 — Supporting File 1: advs74817‐sup‐0001‐SuppMat.pdf. [file ADVS-13-e14972-s002.pdf]

Supplemental Figure 1

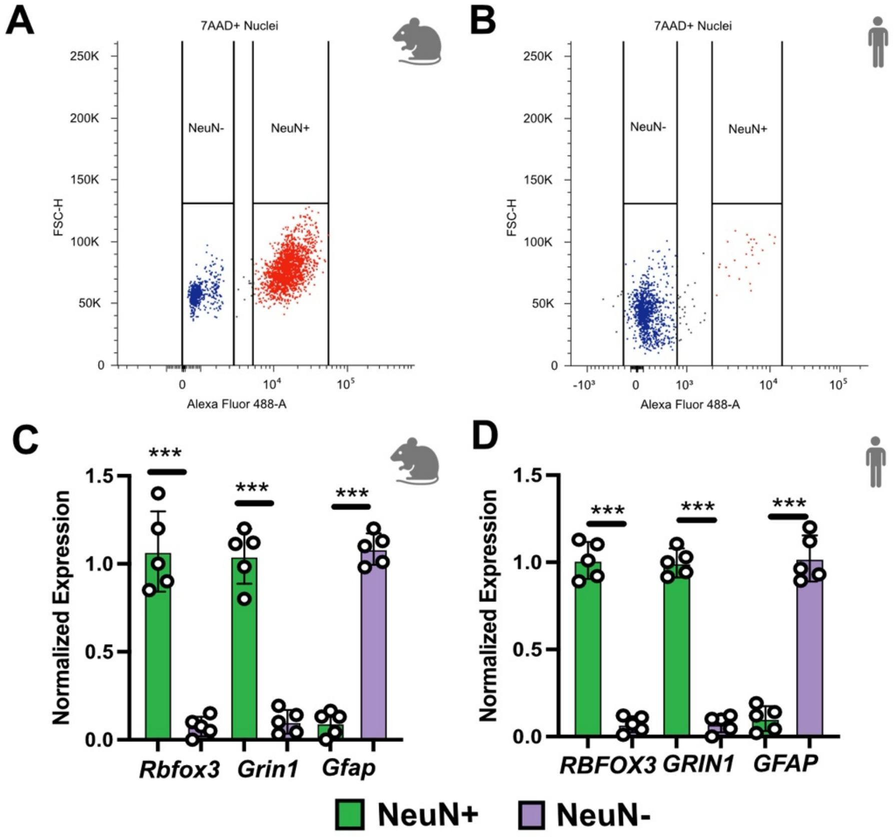

Supplemental Figure 1. FANS sorting strategy for isolation of NeuN$^+$ and NeuN$^-$ nuclei. Fluorescence-activated nuclei sorting (FANS) was performed on isolated nuclei from mouse hippocampus **(A)** and human post-mortem prefrontal cortex tissue **(B)**. For each sample approximately 1,000,000 NeuN$^-$ and NeuN$^+$ nuclei were sorted for mouse. Sorting of human tissue yielded approximately 1,000,000 NeuN$^-$ and 90,000 NeuN$^+$ nuclei (human). The representative FACS plots display 20,000 (mouse) and 60,000 (human) total events per sample. Detected events were gated to exclude debris, doublets, and damaged nuclei. Single nuclei were further gated regarding their 7AAD staining to ensure pure nuclei populations, representing approximately 2.5% of the total events. The 7-AAD$^+$ nuclei was separated into NeuN$^-$ and NeuN$^+$ fractions based on NeuN-Alexa Fluor488 immunoreactivity. The representative images show the final sorting gate for mouse (A) and human (B) NeuN$^-$ and NeuN$^+$ nuclei. Mouse samples yielded robust NeuN$^+$ populations, whereas human post-mortem samples showed a comparatively smaller NeuN$^+$ fraction. This observation is consistent with previous reports and reflects reduced NeuN epitope preservation and increased background fluorescence in post-mortem human nuclei due, rather than the true neuronal composition of the prefrontal cortex. Consequently, FANS gating strategies in human tissue are conservative and prioritize purity of the NeuN$^+$ population over yield. Sorted nuclei populations were subsequently used for downstream single-nucleus RNA sequencing and qPCR analyses. FACS plots were generated using FlowJo. **C.** Bar chart showing qPCR levels of the neuronal marker genes *Rbfox3* and *Grin1*, as well as the astrocyte marker *Gfap*, in nuclei sorted from the mouse hippocampus. $P < 0.001$, unpaired t-test. Data were normalized to NeuN$^+$ nuclei for Rbfox3 and Grin1, and to NeuN$^-$ nuclei for Gfap. **D.** Same as in (C), but sorting was performed from human prefrontal cortex tissue.
FSC-H: Forward Scatter–Height.

Supplemental Figure 2

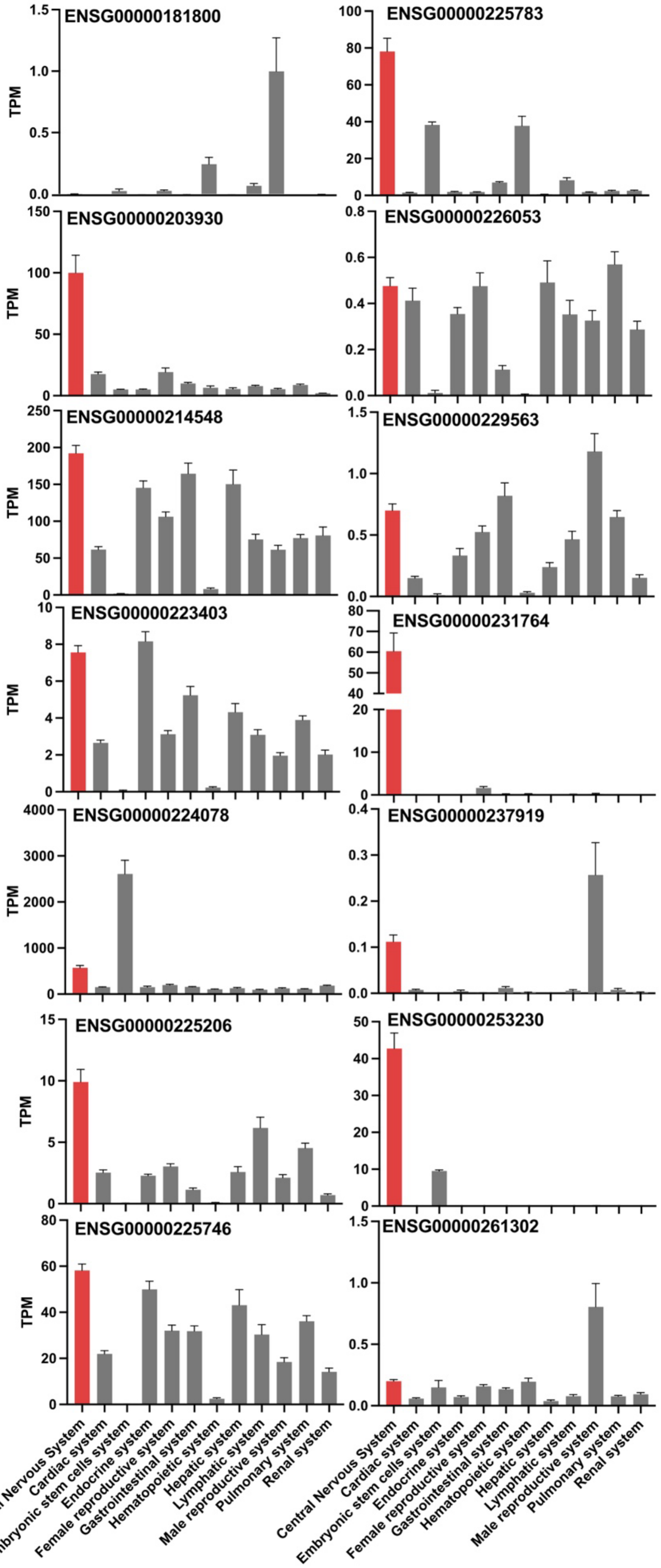

Supplemental Figure 2. Expression of the selected 14 candidate lncRNA (excluding *ENSG00000283183/NEUID* that is shown in Fig 1c, d) across various human tissues obtained from the RNA tissue atlas. TPM; transcripts per million. Expression in the central nervous system is depicted in red.

Supplemental Figure 3

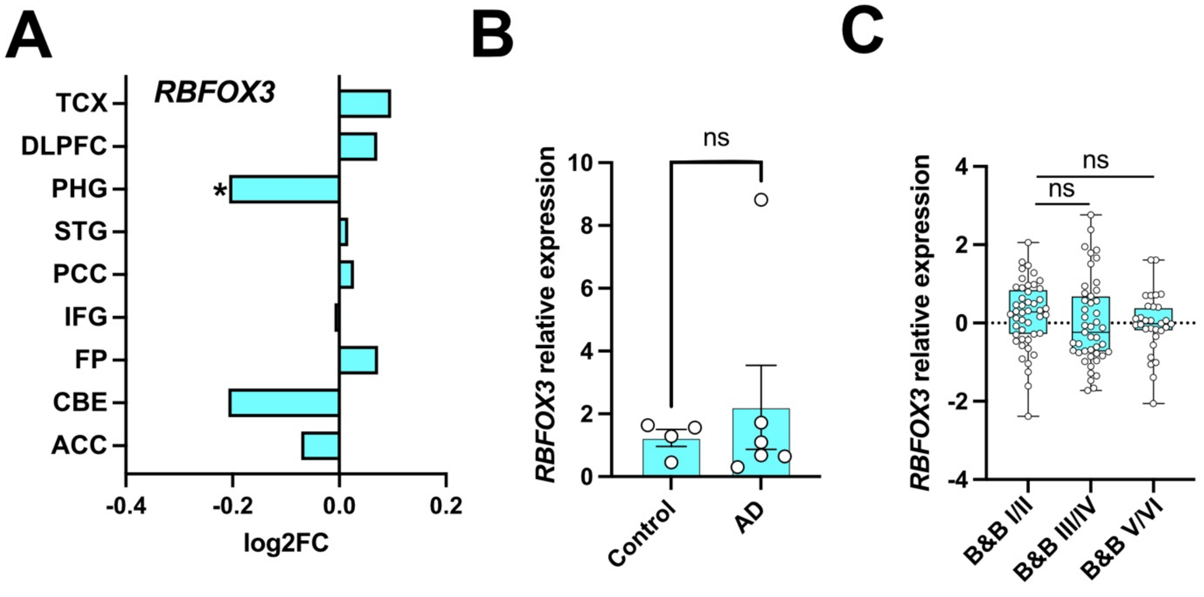

Fig S3. *RBFOX3* expression in the brains of AD patients analyzed for *NEUID* levels. A. *RBFOX3* expression in various brain regions of AD and control postmortem human brains. Data were obtained from the AGORA AD database (*$p < 0.05$). **B.** qPCR analysis of *RBFOX3* expression in prefrontal cortex (Brodmann Area 9, BA9) postmortem brain tissue from control (n = 4) and AD (n = 6) patients. **C.** Expression of *NEUID* in postmortem brain tissue from individuals spanning Braak & Braak (B&B) stages I–VI. Data were obtained from the Framingham Heart Study. One way ANOVA relevant no significant group differences (P = 0,52). Abbreviations: TCX, temporal cortex; DLPFC, dorsolateral prefrontal cortex; PHG, parahippocampal gyrus; STG, superior temporal gyrus; PCC, posterior cingulate cortex; IFG, inferior frontal gyrus; FP, frontal pole; CBE, cerebellum; ACC, anterior cingulate cortex;

Supplemental Figure 4

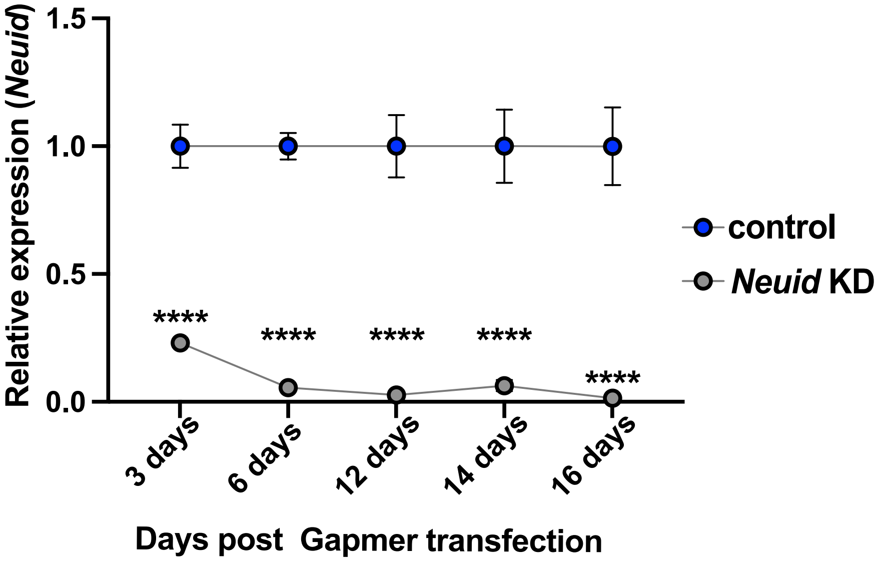

Fig S4. RT-qPCR quantification of *Neuid* expression at indicated time points following administration of *Neuid* Gapmers or control Gapmers in primary hippocampal neurons. One-way ANOVA revealed a significant group difference ($P < 0.0001$, $F = 294.9$); ****$p < 0.001$.

Supplemental Figure 5

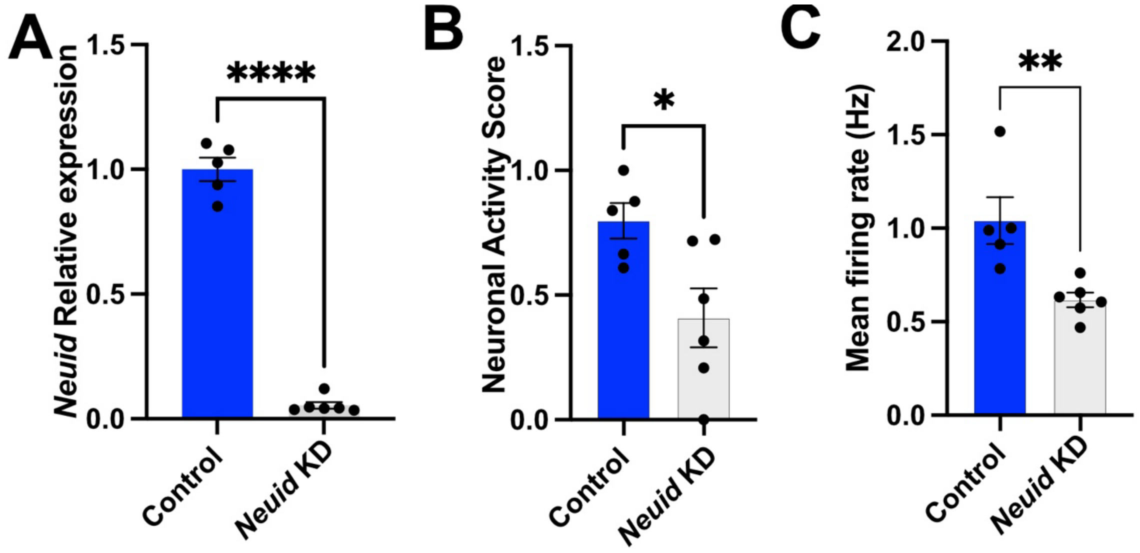

Fig S5. Neural activity in primary cortical neurons upon *Neuid* knockdown. A. Bar plot showing *Neuid* knockdown in primary cortical neurons (n=6). unpaired tTest, ****p < 0.0001. **B.** Bar chart displaying the NAS measured via an MEA assay. *p < 0.05 NeuID KD (n=6) vs control (n=5) ; unpaired tTest. **C.** Bar plot showing the mean firing rate in NeuID KD (n=6) and control (n=5) conditions. **p< 0.01; unpaired tTest.

Supplemental Figure 6

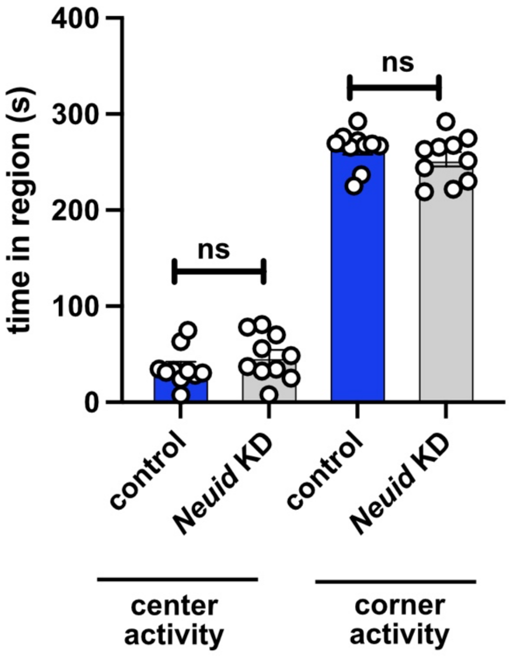

Fig S6. Bar chart depicting time spent in region in open field test between control and *Neuid* Knockdown (KD) groups. The plot shows that both groups spent similar amount of time in the center and the corner, indicating that *Neuid* KD does not induce anxiety-like behavior. (Control, n=9; *Neuid* KD, n=8). ns – not significant, unpaired tTest.

Supplemental Figure 7

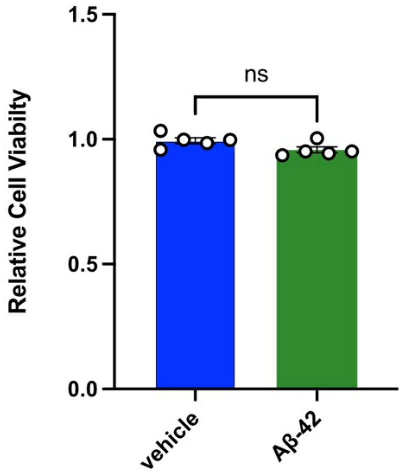

Fig S7. Bar plot showing cell viability of primary hippocampal neurons 24h after Aβ-42 treatment in comparison to treatment with vehicle. ns – not significant, unpaired tTest.

Supplemental Figure 8

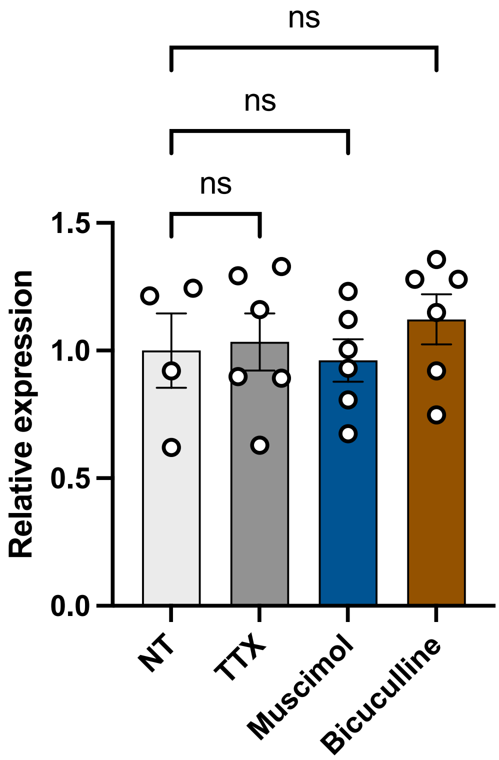

Fig S8. Bar plot showing the relative expression of *Neuid* in primary hippocampal neurons No Treatment (NT, n= 4), Tetrodotoxin (TTX, n = 6), Muscimol (n = 6), and Bicuculline (n = 6); ns-not significant, unpaired tTest.

Supplemental Figure 9

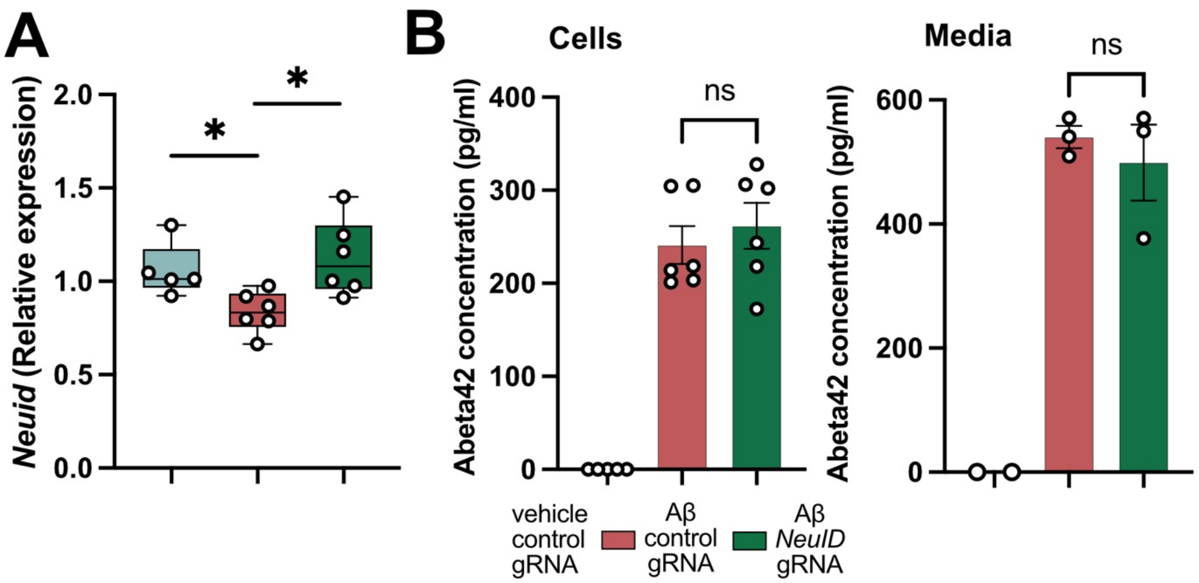

Fig S9. Aβ treatment of CRISPRa mediated Neuid overexpressing primary hippocampal neurons. A. Box plot showing the relative expression of *NeuID* in primary hippocampal neurons that were treated with CRISPRa and either a control gRNA + vehicle (vehicle control gRNA, n=4), Aβ42 + control gRNA (Aβ42 control gRNA, n=6) or Aβ42 along with the *NeuID* gRNA (Aβ42 NeuID gRNA, n= 6); *$p < 0.05$, unpaired tTest. **B.** Bar chart showing the effect of Neuid overexpression on Aβ42 levels in the corresponding cell lysates (left) and media (right) measured via ELISA. ns – not significant, unpaired tTest.

Supplemental Figure 10

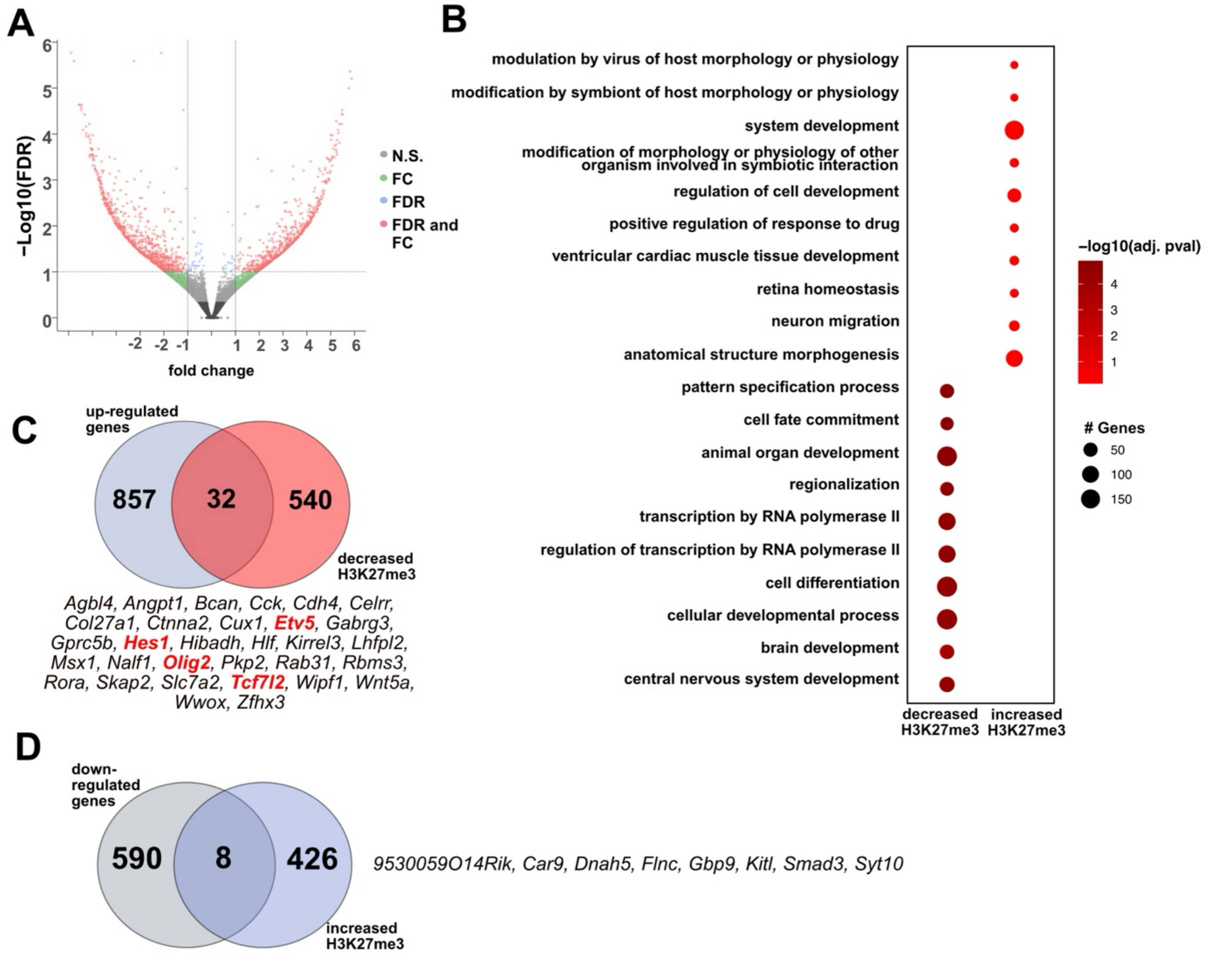

Fig S10. Integrated analysis of H3K27me3 ChIP-sequencing and RNA-sequencing upon *Neuid* KD. A. Volcano plot showing differential H3K27me3 occupancy after *Neuid* KD. Red dots indicate significantly differential peaks/regions assigned to genes (FDR < 0.05, log2 FC > 1). **B** GO enrichment analysis (biological process) for genes associated with regions showing decreased or increased H3K27me3 upon *Neuid* KD. Dot size indicates the number of genes per term, and color indicates significance (–log10 adjusted *P* value). The **top 10 GO terms** for each group are shown. **C.** Venn diagram showing the overlap between genes upregulated in RNA-seq upon *Neuid* KD and genes showing decreased H3K27me3 in ChIP-seq. Thirty-two genes are shared. The corresponding gene list is shown below the Venn diagram, with the four key transcription factors (highlighted in red) that could help explain the increased gene expression observed upon *Neuid* KD (see **Fig. 7H–I**). A GO term analysis of these 32 genes is shown in Fig. 7G. **D.** Overlap between genes downregulated in RNA-seq upon *Neuid* KD and genes showing increased H3K27me3 in ChIP-seq. Eight genes are shared and are listed to the right of the Venn diagram. GO term analysis of these eight genes did not identify any significantly enriched biological processes. Moreover, these genes do not represent known regulators of neurodevelopment. FDR; false discovery rate, N.S., not significant; FC; fold change,

Supplemental Figure 11

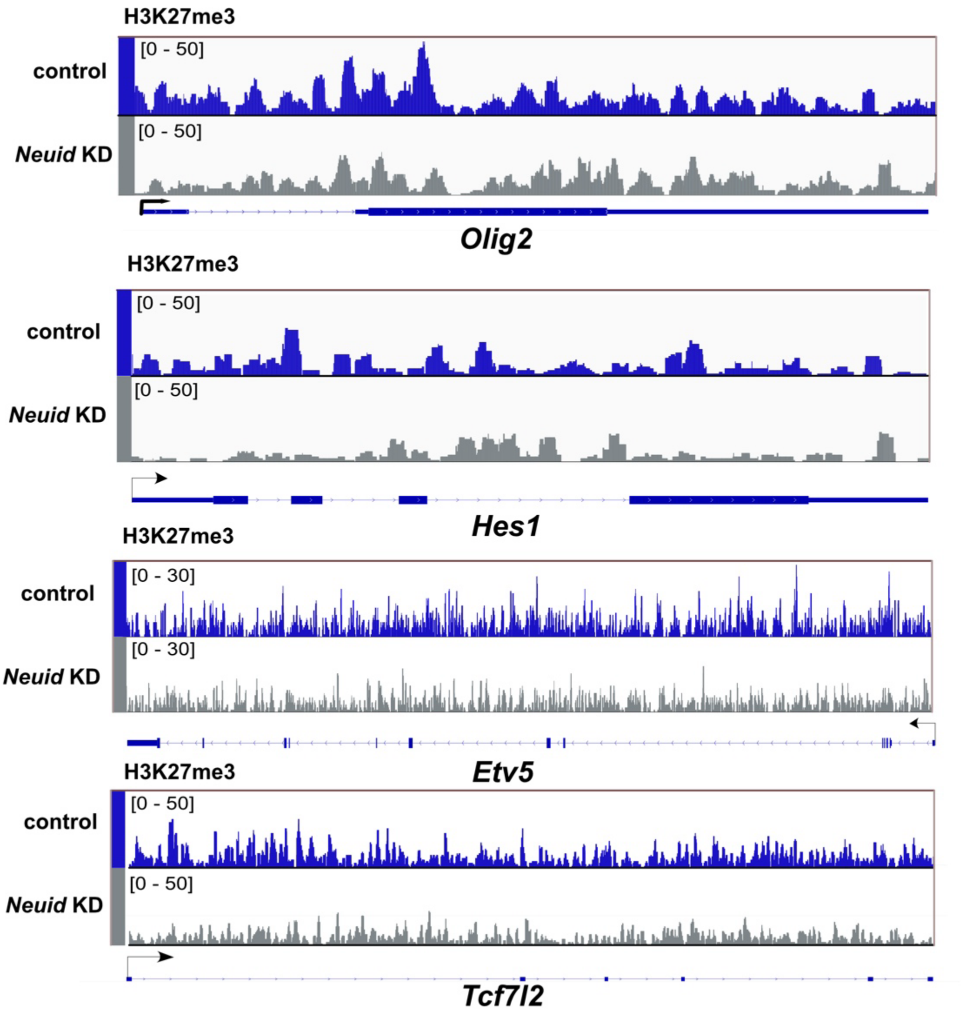

Fig. S11. H3K27me3 levels across key transcription factor loci upon Neuid KD. H3K27me3 ChIP-seq tracks visualized in the Integrated Genomics Viewer (IGV) show decreased H3K27me3 across the loci of four transcription factors *Olig2, Hes1, Etv5, and Tcf7l2* upon *Neuid* KD. Signal intensity is displayed as normalized read coverage using the indicated y-axis scales (0–50 for *Olig2**,** Hes1*, and *Tcf7l2*, and 0–30 for *Etv5*) across conditions. Regions of reduced H3K27me3 extend across the gene bodies, consistent with broad Polycomb-associated repression. Gene models and genomic coordinates are shown below the respective tracks.
